# Exploring Access to Mental Health and Primary Care Services for People With Severe Mental Illness During the COVID-19 Restrictions

**DOI:** 10.3389/fpsyt.2021.799885

**Published:** 2022-01-21

**Authors:** Elizabeth Newbronner, Panagiotis Spanakis, Ruth Wadman, Suzanne Crosland, Paul Heron, Gordon Johnston, Lauren Walker, Simon Gilbody, Emily Peckham

**Affiliations:** ^1^Mental Health and Addiction Research Group, University of York, York, United Kingdom; ^2^Independent Researcher, Clackmannan, United Kingdom

**Keywords:** health services, severe mental ill health, schizoaffecfive disorder, schizophrenia, bipolar disorder, COVID-19, mental health services

## Abstract

**Aims:**

To explore: how satisfied people with severe mental illness (SMI) are with the support received during the pandemic; understand any difficulties encountered when accessing both mental health and primary care services; consider ways to mitigate these difficulties; and assess the perceived need for future support from mental health services.

**Materials and Methods:**

A representative sample was drawn from a large transdiagnostic clinical cohort of people with SMI, which was recruited between April 2016 and March 2020. The sample was re-surveyed a few months after the beginning of the restrictions. Descriptive frequency statistics were used to analyze the quantitative data. The free text responses were analyzed thematically.

**Results:**

367 participants responded to the survey. Two thirds were receiving support from mental health services with the rest supported in primary care or self-managing. A quarter thought they would need more mental health support in the coming year. Half had needed to used community mental health services during the pandemic and the majority had been able to get support. A minority reported that their mental health had deteriorated but they had either not got the supported they wanted or had not sought help. The biggest service change was the reduction in face-to-face appointments and increasing use of phone and video call support. Nearly half of those using mental health services found this change acceptable or even preferred it. However, acceptability was influenced by several factors, and participants were more likely to report that they had received all the support they needed, when seen in person.

**Discussion:**

Although most participants were satisfied with the mental health support they had received, a minority were not. This, couple with findings on future need for mental health support has implications for post pandemic demand on services. Remote care has brought benefits but also risks that it could increase inequalities in access to services.

## Introduction

In mid-March 2020, soon after the WHO declared the outbreak of COVID-19 to be a pandemic, sweeping changes in the way mental health and primary care services in the UK are provided were introduced. A survey of mental health staff, conducted early in the first wave of the pandemic in the UK (i.e., April/May 2020) found that the number of face-to-face meetings was reduced, and phone and video appointments and support were rapidly introduced (1). This observation was supported by a recent report from the House of Lords Covid-19 Committee (2). In most areas of the UK the frequency of contacts and the range of both NHS and third sector services available also reduced (3). Research is beginning to explore the impact of the pandemic and the pandemic restrictions on people with pre-existing mental health conditions. However, there has been little research into the specific experiences of people living with severe mental illness (SMI), and in particular how their access to mental health and primary care services may have changed.

People with SMI are an especially vulnerable group who already experience significant health inequalities. Notably they currently experience a mortality gap of 15–20 years when compared to people without SMI (4–6). An important driver for this gap is preventable physical health conditions, linked to behavioral risk factors, such as poor diet and smoking. However, access to and take-up of services is also a factor (7, 8). There are concerns that the changes in the way services have been delivered during the pandemic may have further increased the barriers to access that people with SMI experience (e.g., access to digital technologies or familiarity/confidence in using them) (9, 10). It is therefore important to: explore how satisfied people with SMI are with the support they received during the pandemic; understand any difficulties they encountered when accessing both mental health and primary care services; consider ways to mitigate these difficulties; and assess the perceived need for future support from mental health services. The aim of this study was to use a large clinical cohort of people with SMI, which was recruited in the years immediately prior to the pandemic restrictions and was re-surveyed a few months after the beginning of the restrictions, to explore these issues.

## Materials and Methods

The Closing the Gap (CtG) Health and Well-being study is a large (*n* = 9,914) observational health study designed to understand the social, behavioral, environmental and economic determinants of health among people with SMI. recruited between April 2016 and March 2020. It comprises adults (aged 18 years or older), recruited between April 2016 and March 2020, who have a documented diagnosis of schizophrenia or delusional/psychotic illness (ICD 10 F20.X & F22.X or DSM equivalent) or bipolar disorder (ICD F31.X or DSM equivalent). Ethical approval for the CtG study was granted by West Midlands—Edgbaston Research Ethics Committee (REF 15/WM/0444).

The OWLS Study, which is a longitudinal study, recruited a sub-cohort from CTG, to explore the effects of the COVID-19 pandemic restrictions on people with severe mental ill health. The paper presents results from the first survey in the study. To be eligible for invitation to OWLS, CtG participants had to have provided contact details and consented to be contacted again, as well as been originally recruited from a clinical site that had the capacity to collaborate with the University of York research team in a new research project. Eligible participants were then organized in groups based on age, gender, ethnicity, and care setting (primary or secondary mental health care) to ensure representation across many sociodemographic groups. From each group, researchers selected a purposive sample of participants that had most recently participated in the CtG study (e.g., recruited in the last 2 years) ensuring that a range of localities was covered. Recent participation to the CtG was considered important to increase response rates (e.g., the team having current and valid contact details, and participants being familiar with the research team). Locality was used to provide geographical diversity, inviting participants from 17 mental health trusts and six Clinical Research Network (CRN) areas in England, including a mix of rural and urban settings.

People who met the eligibility criteria were contacted by telephone or letter and invited to take part in the OWLS COVID-19 study. Those who agreed to take part were offered three options; (i) complete the survey over the phone with a researcher (*n* = 206), (ii) be sent a link to complete the survey online (*n* = 121) or (iii) be sent a hard copy of the questionnaire in the post to complete and return (*n* = 40). Participants were recruited to the OWLS study between July and December 2020.

The full OWLS 1 questionnaire, which is shown in the Supplementary Materials S1, comprised eight sections and included both standardized measures and bespoke questions. The survey was co-designed in conjunction with our Lived Experience Advisory Panel (LEAP) along with input from mental health service providers. Two members of the research team developed an initial draft of the questionnaire. This was then revised following several rounds of comments from the whole research team, LEAP members and health service partners. In this article we focus on the second section of the survey, where we explored how participant's access to mental health and primary care services had changed during the pandemic. For example, we asked participants who were receiving support from mental health services, whether they had experienced one or more specific change (e.g., seeing a different mental health worker to the person they normally saw). If they had, they could indicate how they felt about the change with one of three answers (i.e., I like it better; its ok–not better or worse; or I don't like it). For all services, we were interested to understand how changes in the way services were provided, in particular the shift to phone and online meetings and appointments, might have affected people's experiences, including the extent to which they felt their needs had been met. For mental health services we also asked people about: the support they anticipated needing from services in the year ahead, an issue of particular interest to NHS providers; and how confident they felt about support being available should they need it (an issue which our LEAP felt it was important to explore).

The study analysis plan was registered on Open Science Framework (available at https://doi.org/10.17605/OSF.IO/E3KDM - section 2.1). Descriptive frequency statistics (Ns and percentages) were used to describe key sample characteristics and service use variables. 2 x 2 cross-tabulations and chi-square tests were used to examine the association between (a) unmet need for support and changes in mental or physical health, and (b) mode of service delivery and service satisfaction. The latter was examined for each service type separately (GPs, Community mental health, Mental health crisis services) and *p*-values were corrected for multiple testing (p multiplied by four) using the Bonferroni correction (11). The statistical significance criterion used for all analyses was *p* < 0.05. Analyses were undertaken using SPSS v.26.

In the pre-registered analysis plan it was stated that mode of service delivery would be coded at three-levels (in person, over the phone, or online e.g., video call). However, too few participants reported receiving services online (N ranged from 2 to 14, depending on service type) to allow for meaningful comparisons, and so this was merged with receiving services over the phone into a remote delivery category. The plan also stated that association between Community mental health service satisfaction and mode of service delivery would be stratified per primary or secondary care patients. As very few participants currently in primary care reported getting support from Community mental health services (*N* = 24) to allow for meaningful comparisons, analysis was conducted only in the full sample.

At the end of the survey there was a free text box where respondents could add comments about any aspect of their experiences during the pandemic. 147 (40%) respondents chose to do so. These free text responses we transferred to NVivo and then analyzed thematically (12). The sections of the survey provided the initial structure for the analysis. Comments were then coded inductively within this structure. Fifty-two respondents added comments which in some way related to their access to or experience of using services. We do not present the full thematic analysis in this paper but do use quotations from it to illustrate or bring to life the findings from the quantitative data.

The authors assert that all procedures contributing to this work comply with the ethical standards of the relevant national and institutional committees on human experimentation and with the Helsinki Declaration of 1975, as revised in 2008. All procedures involving human subjects/patients were approved by the North West–Liverpool Central Research Ethics Committee (reference 20/NW/0276). Written or verbal informed consent was obtained from all subjects/patients.

## Results

In this section we begin by briefly describing the demographic characteristics of the OWLS participants. We then present our findings in relation to participants experiences of accessing both community mental health services and mental health crisis services, and their perceived need for future support from mental health services. We then go on to describe their experiences of using primary care services.

The first survey for the OWLS COVID-19 study, which was conducted between July and December 2020, recruited 367 people from the CtG Cohort. Table 1 describes the socio-demographic characteristics and diagnosis of OWLS participants. It should be noted that it was an ethical requirement of the CtG study that the participants consent to their diagnosis being provided to the research team and some participants did not consent to this. In the interests of inclusivity and because there may be some differences between those who consented to their diagnosis being provided and those who did not, we did not make it a requirement of OWLS that the participants should consent to their diagnosis being provided. The mean age was 50.5 (range = 20 to 86, SD ± 15.69) with 51.0% male and 77.4% white British.

**Table 1:** Demographic characteristics of OWLS COVID-19 study SMI population during pandemic restrictions.

**Characteristic**	**Number (%)**
	**Total *N* = 367**
**Gender**	
Male	187 (51.0)
Female	174 (47.4)
Transgender	6 (1.6)
**Age** (mean, range)	50.5 (20–86)
**Ethnicity**	
White British	284 (77.4)
Asian British/Asian	24 (6.5)
Other white	18 (4.9)
Black British/Black	15 (4.1)
Other non-white	12 (3.3)
Mixed white/black	5 (1.4)
Mixed white/Asian	5 (1.4)
Other mixed	4 (1.1)
**Index of deprivation**	
Very high deprivation	97 (26.4)
High deprivation	81 (22.1)
Moderate deprivation	67 (18.3)
Low deprivation	55 (15.0)
Very low deprivation	52 (14.2)
**Diagnosis**	
Psychosis	188 (51.2)
Bipolar	108 (29.4)
Other SMI	23 (6.3)
Not recorded	48 (13.1)

*Some percentages might not add up to 100% due to missing values*.

Of the 367 people who completed the survey, 224 (61%) were receiving support from mental health services, with the rest being supported in primary care or self-managing.

### Community Mental Health Services

Just over half of the survey respondents (194/52.9%) had needed to used community mental health services during the pandemic and the overwhelming majority reported that they had been able to get support. The quotation below highlights the creative approaches sometimes adopted by mental health staff to maintain support:

“*My CPN* [Community Psychiatric Nurse] *was very supportive and has organized my Clozaril bloods to be taken in my garden so I don't need to travel all the way to [TOWN]. I receive regular telephone calls from my CPN and face to face visits every 2 to 3 weeks.”*

We examined whether there was any difference between those who said that their mental health had deteriorated and those who said it had not, in terms of getting support. As Table 2 below shows there was no significant association between deterioration in mental health and receiving mental health services ($\chi_{\left(1\right)}^{2}$ = 0.99, *p* = 0.319). However, 14.5% (*n* = 12) did report that their mental health had deteriorated but they had either not got the supported they wanted or had not sought help. One of the free text comments illustrates this: “*Before the pandemic I wasn't using MH services but as my MH has gotten worse then I have become very conscious of not being able to access services.”*

**Table 2:** Change in mental health and support from community mental health services.

**Mental health change since beginning of pandemic**	**Did you get support from community mental health services?**		
	**Yes (*N* = 164)**	**No/Haven't tried (*N* = 22)**	**Total**
Deterioration	71 (85.5%)	12 (14.5%)	83
No deterioration	93 (90.3%)	10 (9.7%)	103

*8 missing. Percentages are per row*.

Our Lived Experience Advisory Panel suggested that for many people with mental health needs, having confidence that support would be forthcoming if required, was important for people's well-being. So, we asked all survey respondents how confident they felt about support being available, should they need it. More than half (207/56.4%) felt they would be able to access help, but a substantial minority (158/43.1%) were not confident that support would be available.

### Changes to the Way Mental Health Services Were Provided

The pandemic led to substantial and rapid changes in the way in which mental health services were provided. The biggest shift was the reduction in face-to-face appointments and increasing use of phone and online consultations and support. However, people also experienced less frequent contact with services, or had a more limited range of services (including community and voluntary sector services) available to them. A few people saw a different mental health worker. We asked the people who were currently receiving support from mental health services (*n* = 224) how they felt about these changes and 221 provided responses. Table 3 provides and overview of their responses.

**Table 3:** Views about change in the way mental health services were provided.

	**I have not experienced this change**	**I like it better**	**Its ok–not better or worse**	**I don't like it**
I had support on the telephone/online instead of face-to-face appointments	44 (19.9%)	32 (14.5%)	74 (33.5%)	71 (32.1%)
I had less frequent contact with mental health services	105 (47%)	12 (5.4%)	59 (26.7%)	45 (20.4%)
I had more frequent contact with mental health services	147 (67%)	19 (8.7%)	41 (18.8%)	11 (5%)
I had a more limited range of services or support	104 (47.7%)	9 (4.1%)	45 (20.6%)	60 (27.5%)
I saw a different mental health worker to the person who would normally support me	149 (68.7%)	9 (4.1%)	40 (18.4)	19 (8.8%)
I went to a different place for appointments or support	170 (79.1%)	8 (3.7%)	22 (10.2%)	15 (7%)

*Percentages are per row*.

The results show that despite the impact of the pandemic, a high proportion of those survey respondents who were in contact with mental health services, were seeing the same mental health worker, often in the same place. Clearly many people were being supported on the telephone or online instead of face to face, and whilst almost a third did not like this change, nearly half found it acceptable or even preferred it.

The findings from the OWLS study in relation to access to digital devices are reported in a separate paper. However, it is useful to note here that the majority of study participants did have access to a smartphone, tablet, laptop and/or desktop computer. However, 13.4% *(n* = 49) did not have any of these devices. The free text comments also suggested that even where people did have access to such devices, they may not have exclusive use (e.g., a “family” computer), their internet access may be limited or there may be practical and emotional concerns associated with online appointments.

Focusing specifically on the 168 people who had only received support from community mental health services, just over half (*n* = 89/52.7%) had received that support in person. Sixty-six people (39.1%) had support over the phone and a minority (*n* = 14/3.8) had some kind of online video support (e.g., NHS Attend Anywhere). Almost two thirds (*n* = 112/65.9%) were completely satisfied with the support they received but as Table 4 below shows, there was a significant association between satisfaction with the support received from community mental health services and the way in which it was provided ($\chi_{\left(1\right)}^{2}$ = 22.92, *p* < 0.001). Specifically, the proportion of those who reported receiving support in person and were completely satisfied was significantly higher that the proportion of those who reported receiving support remotely and were completely satisfied.

**Table 4:** Satisfaction with support from community mental health services.

**Did you receive the level of support you needed from your community mental health services?**	**How was your appointment delivered?**	
	**In person (*N* = 89)**	**Phone/Online (*N* = 79)**
Completely	73 (82.0%)	37 (46.8%)
Partly/No	16 (18.0%)	42 (53.2%)

*Percentages are per column*.

Many of the free text comments added by respondents were concerned with the shift to phone and online support, including practical difficulties this created:

“*I have had a referral back to mental health services, and I am due to have a phone appointment with a doctor. I will have to find a quiet place to go during work time which will not be easy and I'm worried that I might be overhead. The last appointment was 3 h later than scheduled so I am also worried that it will conflict with other appointments or meetings I have that day.”*

People also highlighted the importance of having an established relationship with their mental health professional: “*Fortunately, I got to know my new CPN through a few face-to-face visits and built a rapport so that now that we speak online it is okay - without the face-to-face it would have been more difficult.”* Others emphasized how much they valued face to face contact:

“*I have had 3 appointments with my psychiatrist over the phone during the lockdown and found this to be a poor substitute compared to a face-to-face meetings. At first my CPN called me once a week for about a 20 min phone call then we used Attend Anywhere the NHS video calling service. Finally, for the last month of the previous lockdown we met in the garden of the local CMHT under a gazebo wearing masks and social distancing. It was great to see my CPN again after so many months and gave me a real boost.”*

Interestingly, a small number of people said they preferred the option of phone and online support, finding it more convenient or less stressful: “*I don't need to be stressed about going out or on the bus, things that make my paranoia and voices worse. And I don't have to leave my dog on her own.”*

### Mental Health Crisis Services

Almost a fifth of respondents (*n* = 59/16.1%) had needed to use mental health crisis services. Over three quarters of this group (*n* = 45/76.3%) were able to get support but a significant minority (*n* = 14/23.7%) reported that they either did not get the support they needed or did not try to get help. The free text comments suggest that for this group the implications of not being able to access support were potentially very serious:

“*Just before lockdown I was trying to manage without medication. I then hit crisis point over a bank holiday period during lockdown. After several calls to the crisis team I was unable to get any help. It wasn't until a suicide attempt did I start to get help. I have only asked for help twice in seven years, so have never abused the service. The overstretched system was sadly lacking in an extreme time of need.”*

We also explored satisfaction with the way in which support was provided (see Table 5). We found there was no significant association between receiving the level of support needed from mental health crisis services and the way in which this support was provided ($\chi_{\left(1\right)}^{2}$ = 0.07, *p* = 4.000) (i.e., the proportion of those who were completely satisfied with the support they received was broadly similar for the in person and phone/online groups).

**Table 5:** Satisfaction with support from mental health crisis services.

**Did you receive the level of support you needed from you mental health crisis Services?**	**How was your appointment delivered?**	
	**In person (*N* = 20)**	**Phone/Online (*N* = 25)**
Completely	12 (60.0%)	14 (56.0%)
Partly/No	8 (40.0%)	11 (44.0%)

*Percentages are per column*.

### Future Need for Mental Health Services

There is substantial interest within the NHS about the impact of the COVID-19 pandemic on future need for mental health services and we were able to explore this to a limited extent in this study. We asked those people who were not currently getting support from mental health services whether they thought they would need support in the year ahead, and just under a third (*n* = 44/31.7%) thought they would.

For those people who were already being supported by mental health services, we asked how their need for support might change in the year ahead. As Table 6 below shows, just over a quarter thought they would need more support either because their mental health had declined, or they had been putting off dealing with some issues, or they felt the support they had prior to the pandemic was insufficient, and almost a quarter thought they might need more support.

**Table 6:** Perceived need for support from mental health services in the year ahead.

**About the year ahead, which of the following fits you best?**	***N* (%)–out of total *N* = 224**
I think I will need the same of less support–my mental health has improved during the pandemic	37 (16.5)
I don't think the support I need will change–I've been ok during the pandemic.	71 (31.7)
I might need more support–the pandemic has been difficult for me but I am going to see how thing go.	54 (24.1)
I will need more support–during the pandemic my mental health has got worse	32 (14.3)
I will need more support–I put off dealing with some things during the pandemic but now I want to get advice/help with them.	18 (8.0)
I didn't have enough support before the pandemic and I still need more support	10 (4.5)

*This question was only asked of participants who were currently getting support from mental health services*.

### Primary Care Services

Two thirds of the survey respondents (*n* = 240) had needed to use their GP practice services during the pandemic (for mental and/or physical health conditions). Of this group, the majority (*n* = 207/86%) reported that they had been able to get an appointment and most (*n* = 154/74.4%) felt they had received the care and support they needed. However, like mental health services, GP practices also moved to much greater use of phone and video consultations. We examined whether there was any association between deterioration in physical health and access to GP care and support. Table 7 below shows that there was no significant association ($\chi_{\left(1\right)}^{2}$ = 0.14, *p* = 0.706).

**Table 7:** Changes in physical health and support from GP services.

**Physical health change since beginning of pandemic**	**Did you get support from GP services?**		
	**Yes (*N* = 202)**	**No/Haven't tried (*N* = 29)**	**Total**
Deterioration	77 (38.1%)	10 (34.5%)	87
No deterioration	125 (61.9%)	19 (65.5%)	144

*Percentages are per row*.

We then examined whether people felt they had received the support they needed. Almost three quarters reported that they had but there was a marked different between those who had had a face-to-face appointment and those supported on the phone or online. As Table 8 shows, people were more likely to feel completely satisfied with the support they received when it was face-to-face ($\chi_{\left(1\right)}^{2}$ = 10.46, *p* = 0.008).

**Table 8:** Satisfaction with support from GP services.

**Did you receive the level of support you needed from your GP?**	**How was your appointment delivered?**	
	**In person (*N* = 87)**	**Phone/Online (*N* = 119)**
Completely	75 (86.2%)	79 (66.4%)
Partly/No	12 (13.8%)	40 (33.6%)

*Percentages are per column*.

In relation to medication and pharmacy services, most survey respondents (*n* = 330/89%) had needed prescription medication during the pandemic and almost all were able to obtain their medication from their usual pharmacy. However, the free text comments revealed that a few people had experienced delays and difficulties with the supply of their usual medication. One respondent said that they were on three types of medication, and at one point had to go to three different pharmacies to get the medication they needed. They noted that they were relatively well but felt that if they had not been, they might have stopped taking their medication. Another explained:

“*There was a shortage of medication supply in the area. I struggled to get my prescription tablets and when I eventually got them then I got a slightly different type (tablets vs. capsules). I wasn't sure what would replace them and how I would deal with them-at the time I thought they were going to give me a different drug and the ones I take are quite a fine balance. So that was very worrying.”*

## Discussion

Most participants reported that they had been able to access support from community mental health services and a relatively high proportion said they were satisfied with the support they received. This contrasts with the findings from a coproduced qualitative interview study by Gillard et al. (10), which involved 49 people “who self-identified as having experiences of mental health difficulties that preceded the pandemic” (p3), not specifically SMI. In this study participants (who were from ethnically diverse background and predominantly living in London) reported inadequate access to mental health services, including issues around continuity of care, not getting treatment as usual and service changes. However, in our study a minority did report that their mental health had deteriorated but they had either not got the supported they wanted or had not sought help. This is an issue of concern, which alongside our findings on future need for increased support, may have implications in terms of post-pandemic increased demand for mental health services. Furthermore, for some people living with severe mental illness, the fear of becoming unwell and not being able to access support is a source of anxiety. A substantial minority of participants in our study did not feel confident that support would be available should they need–a perception that mental health services may need to explore and address.

The biggest change in the way in which mental health services were provided was the reduction in face-to-face appointments and the increase in remote care. We found that almost half those who had used community mental health service had received support in person, with staff using creative approaches to maintaining face-to-face contact. Many people were being supported on the telephone and a few had online support. Whilst almost a third did not like this change, nearly half found it acceptable or even preferred it. However, the free text comments suggest that acceptability was influenced by several factors. In particular, respondents felt that telephone or video appointments were far easier when: they had an established relationship with their mental health professional; where they had somewhere private to hold a telephone conversation; or they had access to a (private) digital device-findings which echo those of a recent qualitative study by Liberati et al. (13). Furthermore, whilst increased use of remote mental health care might be acceptable for a limited period, during a public health crisis, in the long term it could lead to problems with accessibility, equity of care and service quality (9, 10, 13). The limited availability of online support reported by participants in our study, suggests that Greenhalgh et al. (14) are correct in suggesting that the evidence relating to video-based remote care needs to be strengthened. Interestingly, whilst people were more likely to feel that they had received all the support they needed from community mental health service when they had been seen in person, in mental health crisis services the way support was provided appeared less important. We speculate that this could be because phone support is already commonly used in crisis services and/or because remote care facilitated more rapid access to support.

With regard to access to primary care services, the majority of participants had been able to get an appointment and most felt they had received the care and support they needed. However, those who had a face-to-face appointment were more likely to report they had received the support they needed. Interestingly, the free text comments revealed a specific issue in relation to obtaining medication, with a number of respondents reporting delays and difficulties with the supply of their usual medication.

Overall then, our study found that the majority of people with SMI were able to access support but those who received remote support were less likely to be satisfied with that support. Furthermore, the context of remote support was important, a finding supported in a recent study by Vera San Juan et al. (15). This suggests that if, as seems likely, service providers continue to employ some element of remote care, service users should have a choice about whether they want remote care and the mode of remote support they receive. It may also be important to ensure some core community services are provided face to face. More broadly, as services continue to adapt to the prevailing pandemic situation and review future provision, there should be a renewed focus on the implications of intersectionality. Specifically, how the experience of living with severe mental illness, coupled with other vulnerabilities or disadvantages such as disabling physical conditions, low income, and ethnicity can create even greater inequalities in access to services.

The findings from this study suggest that some people with SMI do not feel that they have received enough support during the pandemic, or have been reluctant to seek support, perhaps because of the way services were provided or fear of contracting the virus. As a consequence, when the pandemic restrictions ease there could be an increase in demand from mental health services from existing service users. However, this may be tempered by the fact that many mental health professionals and GPs appear to have prioritized support for people with SMI and found creative ways to maintain some face-to-face contact.

Some of the changes introduced as a result of the COVID-19 pandemic will almost certainly continue to be part of future mental health and primary care service. These changes can bring benefits for service providers and some service users. However, they could also increase the risk of unequal access to support and poorer quality of care. To counter these risks, people with SMI must be consulted about the nature of any long-term changes, and going forward, close attention needs to be paid to services user's individual circumstance and preferences.

## Strengths and Limitations

This study focuses on the experiences of people with severe mental illness and as such is an important addition to wider literature about the impact of the pandemic on people with pre-existing mental health conditions. The relatively large and diverse sample, drawn from across England, allowed us to explore a range of experiences. However, by their nature surveys do not enable in depth examination of topics, or exploration of the reasons behind certain findings (e.g., the low availability of video calls). We also recognize that during the survey period there were changes on the nature and level of pandemic restrictions, which were not able to take account of in our analysis.

## Data Availability Statement

The raw data supporting the conclusions of this article will be made available by the authors, without undue reservation.

## Ethics Statement

The studies involving human participants were reviewed and approved by North West–Liverpool Central Research Ethics Committee (reference 20/NW/0276). Written informed consent for participation was not required for this study in accordance with the national legislation and the institutional requirements.

## Author Contributions

EP, PS, PH, SC, EN, LW, and RW contributed to the design of the survey. PS, PH, SC, and LW administered the survey to participants and collected data. EP, PS, and PH cleaned and organized the dataset. EN, RW, EP, PS, and PH conducted the analyses. EN wrote the manuscript. GJ provided guidance from a lived experience perspective. SG provided senior academic guidance. All authors contributed to the conception and design of the study, the interpretation of the findings, manuscript revision, read, and approved the submitted version.

## Funding

This study was supported by the Medical Research Council (Grant No. MR/V028529) and links with the Closing the Gap cohort, which was part-funded by the Wellcome Trust (reference 204829) through the Centre for Future Health at the University of York, UK Research and Innovation (reference ES/S004459/1), and the NIHR Yorkshire and Humber Applied Research Collaboration.

## Author Disclaimer

Any views expressed here are those of the project investigators and do not necessarily represent the views of the Medical Research Council, Wellcome Trust, UK Research and Innovation, National Institute for Health Research or the Department of Health and Social Care.

## Conflict of Interest

The authors declare that the research was conducted in the absence of any commercial or financial relationships that could be construed as a potential conflict of interest.

## Publisher's Note

All claims expressed in this article are solely those of the authors and do not necessarily represent those of their affiliated organizations, or those of the publisher, the editors and the reviewers. Any product that may be evaluated in this article, or claim that may be made by its manufacturer, is not guaranteed or endorsed by the publisher.
