# Supplementary material for: Exploring Access to Mental Health and Primary Care Services for People With Severe Mental Illness During the COVID-19 Restrictions
Source: Front Psychiatry. 2022 Jan 21;12:799885. doi: 10.3389/fpsyt.2021.799885 (PMC8814571; doi:10.3389/fpsyt.2021.799885)
Supplement: Supplementary file 2 [file Data_Sheet_2.docx]

**S2 Results for General Hospital Services**

Over a third of the survey respondents (n=137/37%) had needed a routine hospital appointment for a physical health problem. The majority of this group (n=103/75.1%) had been able to get an appointment, with more than half (n=73/53.2%) seeing their health care professional in person. We also examined whether people felt they had received the support they needed. Three quarters (n=79/75.9%) reported that they had but here was a significant association between peoples’ satisfaction with the support they received and the way the appointment was delivered. The proportion of those were completely satisfied was significantly higher for those who had a face-to-face appointment, compared to those who were supported remotely.
